# LC-MS Profiling of Kakkonto and Identification of Ephedrine as a Key Component for Its Anti-Glycation Activity

**DOI:** 10.3390/molecules28114409

**Published:** 2023-05-29

**Authors:** Kaori Ito, Takashi Kikuchi, Kanako Ikube, Kouharu Otsuki, Kazuo Koike, Wei Li

**Affiliations:** Faculty of Pharmaceutical Sciences, Toho University, Miyama 2-2-1, Funabashi 274-8510, Chiba, Japan

**Keywords:** Kampo, Kakkonto, anti-glycation activity, advanced glycation end products, ephedrine

## Abstract

A total of 147 oral Kampo prescriptions, which are used clinically in Japan, were evaluated for their anti-glycation activity. Kakkonto demonstrated significant anti-glycation activity, prompting further analysis of its chemical constituents using LC-MS, which revealed the presence of two alkaloids, fourteen flavonoids, two but-2-enolides, five monoterpenoids, and four triterpenoid glycosides. To identify the components responsible for its anti-glycation activity, the Kakkonto extract was reacted with glyceraldehyde (GA) or methylglyoxal (MGO) and analyzed using LC-MS. In LC-MS analysis of Kakkonto reacted with GA, the peak intensity of ephedrine was attenuated, and three products from ephedrine-scavenging GA were detected. Similarly, LC-MS analysis of Kakkonto reacted with MGO revealed two products from ephedrine reacting with MGO. These results indicated that ephedrine was responsible for the observed anti-glycation activity of Kakkonto. Ephedrae herba extract, which contains ephedrine, also showed strong anti-glycation activity, further supporting ephedrine’s contribution to Kakkonto’s reactive carbonyl species’ scavenging ability and anti-glycation activity.

## 1. Introduction

Protein glycation, which is the non-enzymatic reaction between reducing sugars and proteins, results in the formation of advanced glycation end products (AGEs). In the first step of glycation, reducing sugars react reversibly with free amino groups on the protein through a nucleophilic attack, leading to the formation of Schiff bases. Intermediate products such as 3-deoxyglucosone undergo slow rearrangement to form stable Amadori products or ketoamines [1,2,3,4]. Furthermore, reactive dicarbonyl compounds such as glyoxal and methylglyoxal (MGO), which are primarily produced as by-products of glycolysis, can also cause the formation of AGEs by selectively reacting with the side chains of basic amino acids such as arginine and lysine on proteins [3]. The aforementioned reactive carbonyl species, including glyoxal, MGO, and 3-deoxyglucosone, are highly reactive and represent important precursors of AGEs in the human body [5]. Accumulation of AGEs in tissues leads to the formation of irreversible cross-linked structures with proteins [6], resulting in pathological reactions in tissues and organs and causing a series of diabetic complications [7]. Furthermore, AGEs and reactive carbonyl species are also implicated in aging [8], Alzheimer’s disease [9], and atherosclerosis [10]. While several compounds, including aminoguanidine [11] and metformin [12,13], have been investigated for their ability to inhibit the formation of AGEs and avert diabetic complications, their clinical application has been constrained due to apprehensions regarding their safety and efficacy. Aminoguanidine, for example, failed clinical trials of ACTION II due to its high toxicity in diabetic patients [14]. Hence, it is a critical issue to develop effective and safe anti-AGE drugs to protect people with diabetes from complications.

Kampo medicine is a traditional Japanese medicine that originated in ancient China and underwent its unique development in Japan. There are currently 294 Kampo prescriptions available as over-the-counter (OTC) medications and 148 Kampo prescriptions available as prescription medications in Japan. Kampo medicines are gaining attention as an alternative or complementary treatment option to Western medicines, particularly for chronic conditions that may not be fully addressed using conventional medicine alone. In recent years, there has been increasing research on Kampo medicines, with studies focusing on their efficacy, safety, and mechanism of action [15,16,17]. As part of our ongoing research to scientifically clarify the effectiveness of Kampo medicines [18,19,20,21], this study evaluated the anti-glycation activity of a total of 147 prescriptions of oral Kampo medicines covered by health insurance in Japan. Among them, Kakkonto showed significant activity, and its chemical constituents were further studied using LC-MS analysis. The components responsible for the anti-glycation activity were identified by analyzing the Kakkonto extract that was reacted with glyceraldehyde (GA) or MGO using LC-MS.

## 2. Results and Discussion

### 2.1. Evaluation of Anti-Glycation Activity of 147 Oral Kampo Prescriptions

In Japan, a total of 147 oral Kampo prescriptions are covered by health insurance. To evaluate the anti-glycation activity of Kampo medicines, all of these oral prescriptions were screened using the bovine serum albumin (BSA)–D-ribose assay according to a method in the literature with minor modifications [22]. BSA was incubated with D-ribose to induce glycation, and the formation of AGEs was measured using fluorescence spectroscopy. This assay can be evaluated within a short incubation time of 1 h. Of the Kampo medicines tested, 18 prescriptions, including Kakkonto, exhibited anti-glycation activity of more than 50% inhibition at a concentration of 50 μU/mL (Table 1).

**Figure 1:**
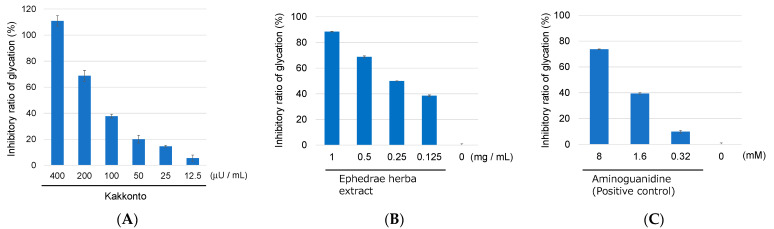
Anti-glycation activity of Kakkonto (**A**), Ephedrae herba extract (**B**), and aminoguanidine (positive control) (**C**) at different concentrations in the BSA–GA assay.

Subsequently, the anti-glycation activity of the 18 prescriptions was evaluated using a BSA–glyceraldehyde (GA) assay according to the manufacturer’s protocol. GA is a reactive carbonyl species that is produced during sugar metabolism. Out of the 18 prescriptions, 14 showed anti-glycation activity of more than 50% inhibition at a concentration of 400 μU/mL (Table 1). Kakkonto was found to exhibit more than 80% inhibitory activity in both assays and was selected for further study. Further testing of Kakkonto was carried out at different concentrations ranging from 400 to 12.5 μU/mL in the BSA–GA assay. The results showed that Kakkonto inhibited the formation of AGEs in a dose-dependent manner, with an IC_50_ value of 120 μU/mL (Figure 1A).

### 2.2. Identification of Chemical Constituents in Kakkonto by LC-MS

**Figure 2:**
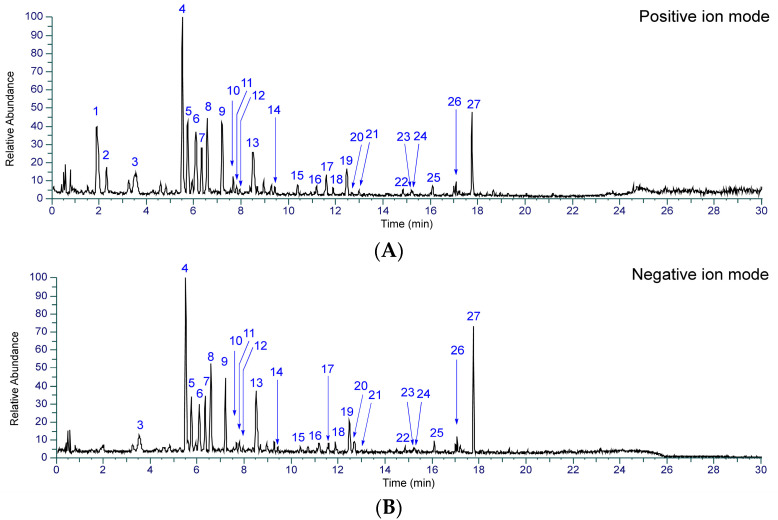
Total ion chromatograms of Kakkonto in positive ion mode (**A**) and negative ion mode (**B**).

**Figure 3:**
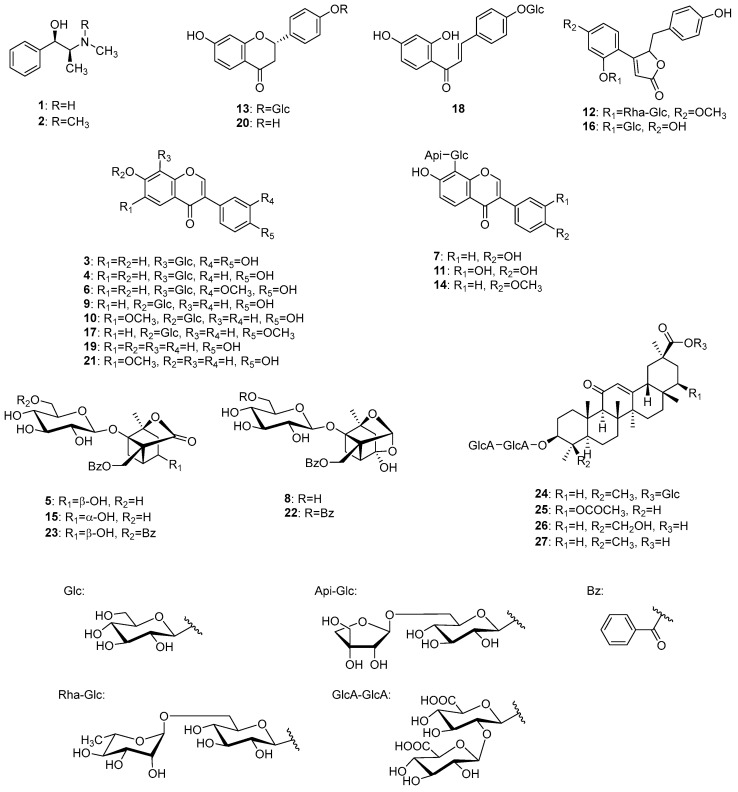
Structures of identified compounds in Kakkonto.

To identify the chemical constituents in Kakkonto, an LC-MS analysis was conducted. As shown in Figure 2, a total of twenty-seven peaks were detected in the total ion chromatogram, and their retention time and MS fragmentation ions are listed in Table S2. By carefully analyzing the chromatographic behaviors and MS data and referring to the literature [23,24,25,26,27], the presence of two alkaloids (**1**, **2**), fourteen flavonoids (**3**, **4**, **6**, **7**, **9**–**11**, **13**, **14**, and **17**–**21**), two but-2-enolides (**12**, **16**), five monoterpenoids (**5**, **8**, **15**, **22**, **23**), and four triterpenoid glycosides (**24**–**27**), were identified (Figure 3). The structure elucidation based on MS fragmentation is described briefly as follows.

**Figure 4:**
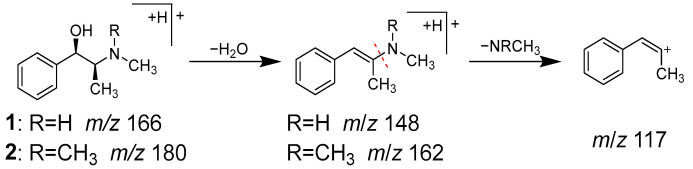
MS/MS fragmentation pathway of compounds **1** and **2**.

Peaks **1** (t_R_ 1.92 min) and **2** (t_R_ 2.32 min) were identified as ephedrine (**1**) and methylephedrine (**2**), respectively, which are commonly found in Ephedrae herba. MS of both compounds exhibited molecular ions [M+H]^+^ at *m/z* 166.1227 and 180.1383, respectively, in positive ion mode, which is consistent with their molecular formulas of C_10_H_15_ON and C_11_H_17_ON. In MS/MS analysis, they revealed a similar fragmentation pattern, with characteristic product ions due to the desorption of H_2_O and the methyl amino or dimethyl amino groups from [M+H]^+^ (Figure 4).

**Figure 5:**
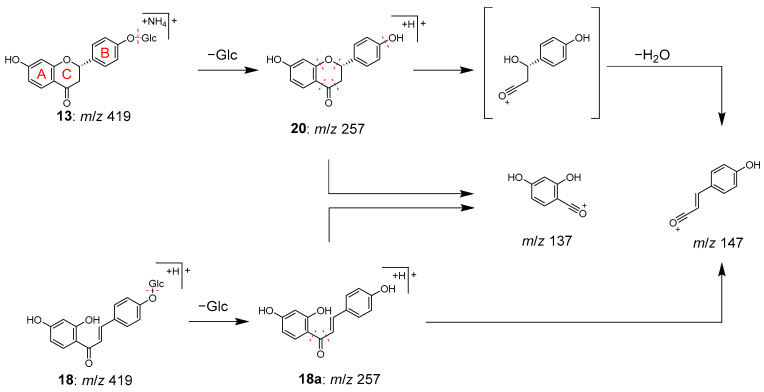
MS/MS fragmentation pathway of compounds **13**, **18**, and **20**.

Peaks **13** (t_R_ 8.50 min) and **18** (t_R_ 11.88 min) were identified as liquiritin (**13**) and isoliquiritin (**18**), and peak **20** (t_R_ 12.71 min) was the aglycone of **13**, which was liquiritigenin (**20**). These compounds are all contained in Glycyrrhizae radix [28]. Peaks **13** and **18** have the same molecular formula of C_21_H_22_O_9_, which was deduced from the ions of *m/z* 436.1597 [M+HN_4_]^+^ and *m/z* 419.1333 [M+H]^+^, respectively. In MS/MS, they also exhibited the same product ions [Aglycone+H]^+^ at *m/z* 257 resulting from the detachment of the glucose moiety, and ions at *m/z* 137 and *m/z* 147 derived from the A ring and B ring, respectively (Figure 5). Although the MS data alone could not distinguish peaks **13** and **18**, they were identified based on the retention times [29]. Similarly, peak **20** was most likely to be the aglycone of **13** or **18**, as evidenced by its molecular formula of C_15_H_12_O_4_ and the MS/MS product ions of *m/z* 137 and *m/z* 147 from the precursor [M+H]^+^ ion. Due to its slightly longer retention time than **18**, peak **20** was identified as liquiritigenin (**20**) instead of isoliquiritigenin (**18a**) [30].

Peaks **3**, **4**, **6**, **7**, **9**–**11**, **14**, **17**, **19**, and **21** were identified as isoflavones (**19**, **21**) and their *O*-glycosides (**9**, **10**, **17**) and *C*-glycosides (**3**, **4**, **6**, **7**, **11**, **14**).

Peaks **19** (t_R_ 12.47 min) and **21** (t_R_ 12.99 min) were identified as daidzein (**19**) and glycitein (**21**), respectively. Peak **19** has been reported to be contained in both Glycyrrhizae radix [31] and Puerariae radix [32], while **20** has been reported to be contained in Puerariae radix [32]. Peaks **19** and **21** have the molecular formulas of C_15_H_10_O_4_ and C_16_H_12_O_5_, which were deduced from the molecular ions of *m/z* 255.0649 [M+H]^+^ and *m/z* 285.0754 [M+H]^+^, respectively.

**Figure 6:**
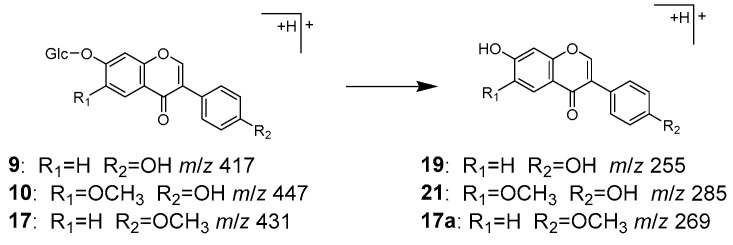
MS/MS fragmentation pathway of compounds **9, 10**, and **17**.

Peaks **9** (t_R_ 7.20 min), **10** (t_R_ 7.68 min), and **17** (t_R_ 11.60 min) were identified as daidzin (**9**), glycitin (**10**), and ononin (**17**), respectively, which are isoflavone *O*-glycosides. Compounds **9** and **17** have been reported to be contained in both Puerariae radix and Glycyrrhizae radix [32,33], while **10** has been found in Puerariae radix [32]. In the MS/MS of [M+H]^+^, the product ions were observed as [Aglycone+H]^+^ ions corresponding to **19**, **21**, and hormononetin (**17a**), resulting from the detachment of the glucose moiety (Figure 6).

Peaks **3** (t_R_ 3.51 min), **4** (t_R_ 5.52 min), **6** (t_R_ 6.10 min), **7** (t_R_ 6.35 min), **11** (t_R_ 7.82 min), and **14** (t_R_ 9.43 min) were identified as 3′-hydroxypuerarin (**3**), puerarin (**4**), 3′-methoxypuerarin (**6**), puerarin apioside (**7**), 3′-hydroxypuerarin apioside (**11**), and formononetin 8-*C*-[β-D-apiofuranosyl-(1→6)]-β-D-glucopyranoside (**14**), respectively, which are characteristic isoflavone *C*-glycosides originated from Puerariae radix [34,35,36,37].

**Figure 7:**
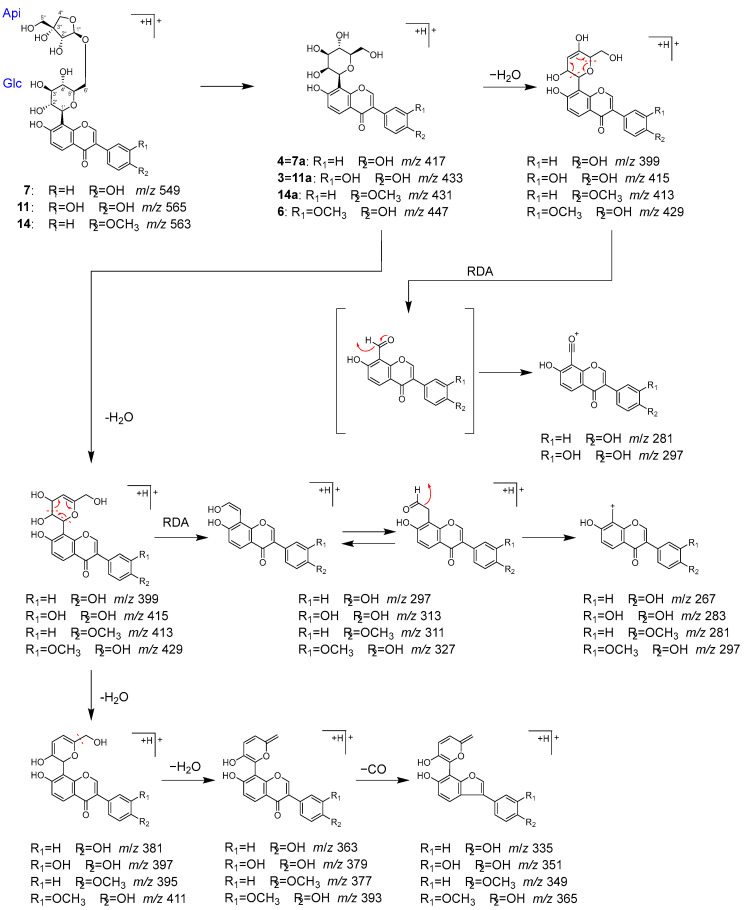
MS/MS fragmentation pathway of compounds **3**, **4**, **6**, **7**, **11**, and **14**.

Compounds **3**, **4**, and **6** were considered to have one sugar attached to the isoflavone based on their molecular formula. These compounds exhibited a series of product ions due to the elimination of H_2_O from the sugar moiety, followed by the characteristic bond cleavage of *C*-glycosidide via retro-Diels–Alder reaction, as well as elimination of hydride, formyl group, H_2_O, and CO. On the other hand, it was revealed that **7**, **11**, and **14** have an apiose moiety bound to **4**, **3**, and 4′-methoxypuerarin (**14a**), respectively, since in MS/MS of **7**, **11**, and **14**, the product ions generated by the detachment of terminal sugar corresponded to **4**, **3**, and **14a** (Figure 7).

Peaks **12** (t_R_ 7.96 min) and **16** (t_R_ 11.19 min) were identified as pueroside A (**12**) and sophoraside A (**16**), respectively, which are characteristic but-2-enolide derivatives of Puerariae radix [38].

**Figure 8:**
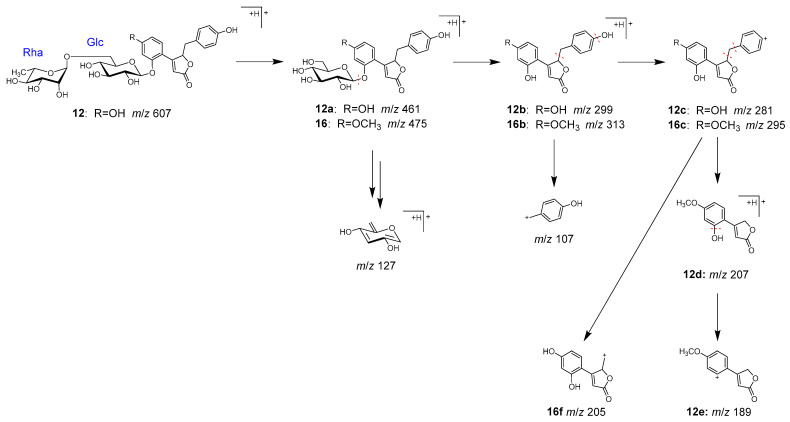
MS/MS fragmentation pathway of compounds **12** and **16**.

In MS/MS, **12** underwent fragmentation to generate fragment ion **12a**, corresponding to a demethylated **16**, through the elimination of terminal rhamnose. Further fragmentation of **12a** and **16** generated **12b** and **16b** by the loss of a glucose, then **12c** and **16c** by the loss of a hydroxy group. Furthermore, **12d** and **12e** were generated from **12c** via the elimination of benzoyl and hydroxy groups, and **16f** from **16c** via the elimination of a phenyl group (Figure 8).

Peaks **5** (t_R_ 5.76 min), **8** (t_R_ 6.57 min), **15** (t_R_ 10.38 min), **22** (t_R_ 14.86 min), and **23** (t_R_ 15.20 min) were identified as characteristic monoterpenoids in Paeoniae radix [39].

**Figure 9:**
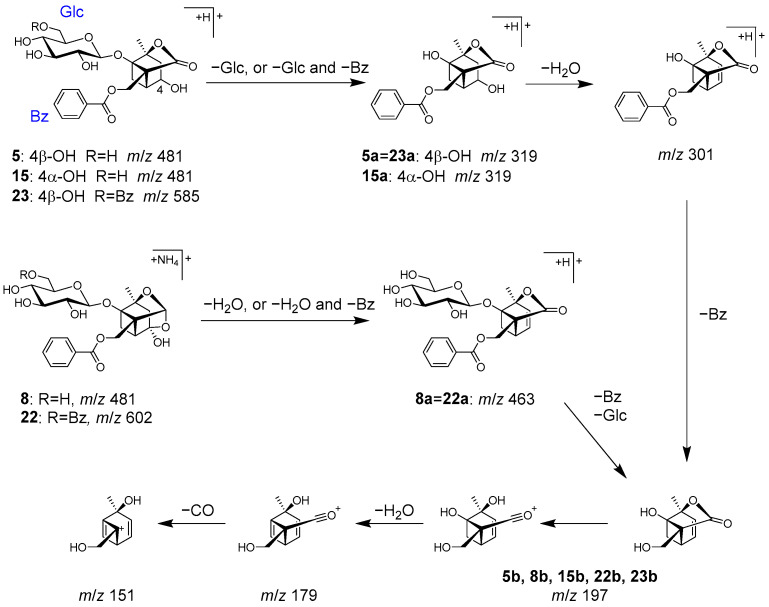
MS/MS fragmentation pathway of compounds **5**, **8**, **15**, **22**, and **23**.

Peaks **5**, **15**, and **8** were identified as albiflorin (**5**), 4-epi-albiflorin (**15**), and peoniflorin (**8**), with the same molecular formula (C_23_H_28_O_11_ each), and they were deduced from the ions of *m/z* 498.1966 [M+NH_4_]^+^, 481.1699 [M+H]^+^, and *m/z* 481.1704 [M+H]^+^, respectively. Fragmentation of **5** and **15** generated ions **5a** and **15a** via the loss of a glucose, followed by the loss of H_2_O and the benzoyl group to generate product ions **5b** and **15b** (Figure 9). On the other hand, peoniflorin (**8**) first produced ion **8a** via the loss of H_2_O, followed by the loss of the benzoyl group and glucose to produce ion **8b**. A series of product ions from **5b**, **8b**, and **15b** were generated by the loss of H_2_O and CO (Figure 9).

Paeonivayin (**23**) and benzoylpaeoniflorin (**22**) possess structures with an additional benzoyl moiety attached at C-6 of the glucose of **5** and **8**, respectively. In MS/MS, product ions from **23** and **22** were observed to be the same as those from **5** and **8** (Figure 9). Notably, **5**, **8**, **15**, **22**, and **23** can also be distinguished by their retention times [26].

Peaks **24** (t_R_ 15.24 min), **25** (t_R_ 16.09 min), **26** (t_R_ 17.08 min), and **27** (t_R_ 17.77 min) were identified as licoricesaponin A_3_ (**24**), 22β-acetoxyglycyrrhizic acid (**25**), licorice saponin G_2_ (**26**), and glycyrrhizin (**27**), which are contained in Glycyrrhizae radix [40].

**Figure 10:**
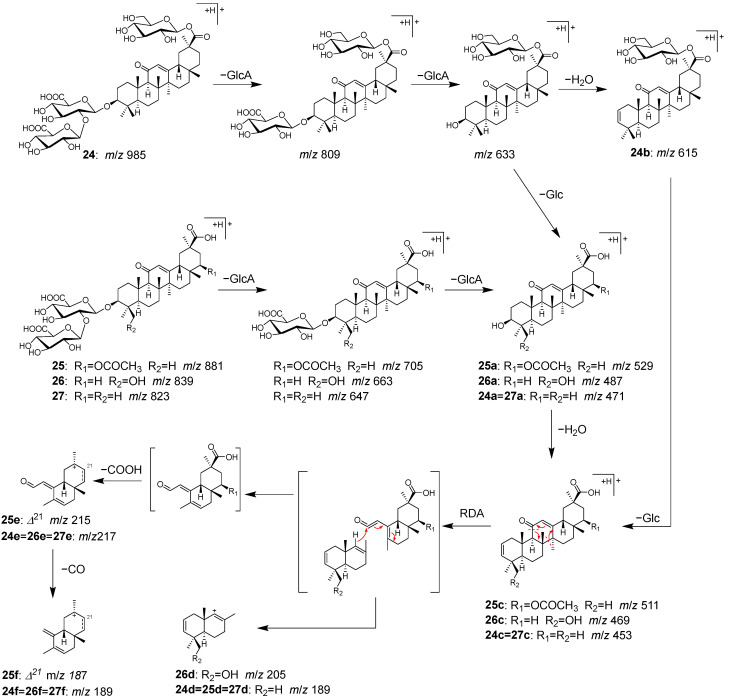
MS/MS fragmentation pathway of compounds **24**–**27**.

Peak **27** exhibited a higher peak intensity and was identified as glycyrrhizin (**27**) based on the observed [M+H]^+^ ion at *m/z* 823.4097, which corresponds to the molecular formula (C_42_H_62_O_16_). In addition, the molecular formulas of **24**, **25**, and **26** were deduced from the [M+H]^+^ ions at *m/z* 985.4637, 881.4155, and 839.4055, respectively. This suggested that **24** (C_48_H_72_O_21_) is a glucosylated **27**, **25** (C_44_H_64_O_18_) is an acetoxylated **27**, and **26** (C_42_H_62_O_17_) is a hydroxyated **27**. In MS/MS of **24**–**27**, consecutive fragmentation generated the product ions of **24a**–**27a** [Aglycone+H]^+^ via deglycosylation and **24c**–**27c** via dehydration. Further fragmentation resulted in the cleavage of the C-ring structure by a Retro-Diels–Alder reaction, which led to the observation of some important ions for structural identification, including **24d**–**27d** derived from the AB ring, and **24d**–**27d** and **24f**–**27f** from the CD ring (Figure 10).

Additionally, LC-MS analysis revealed that Kakkonto contains ephedrin (**1**), puerarin (**4**), albiflorin (**5**), 3′-methoxypuerarin (**6**), puerarin apioside (**7**), paeoniflorin (**8**), daidzin (**9**), liguiritin (**13**), ononin (**17**), daidzein (**19**), and glycyrrhizin (**27**) in relatively high contents.

### 2.3. Identification of the Components That Contribute to the Anti-Glycation Activity

**Figure 11:**
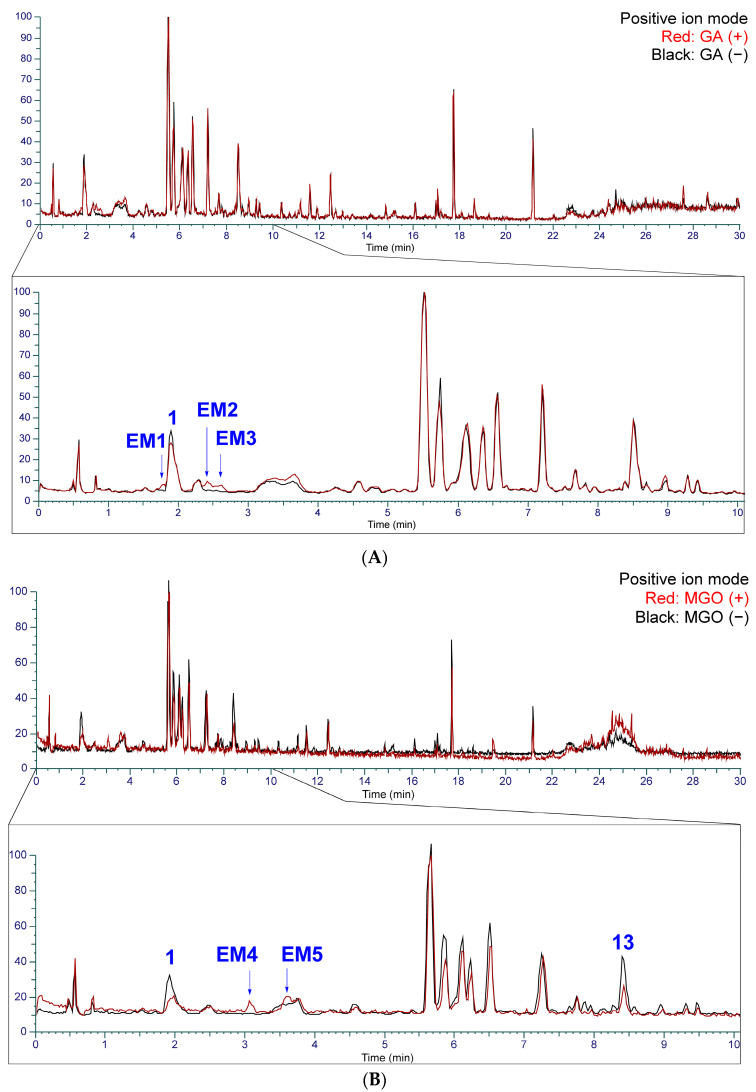
The total ion chromatogram of Kakkonto reacted with glyceraldehyde (GA) and methyl glyoxal (MGO) in positive ion mode. (**A**) Superposition of Kakkonto extract reacted with GA (red) and without GA (black). (**B**) Superposition of Kakkonto extract reacted with MGO (red) and without MGO (black).

**Figure 12:**
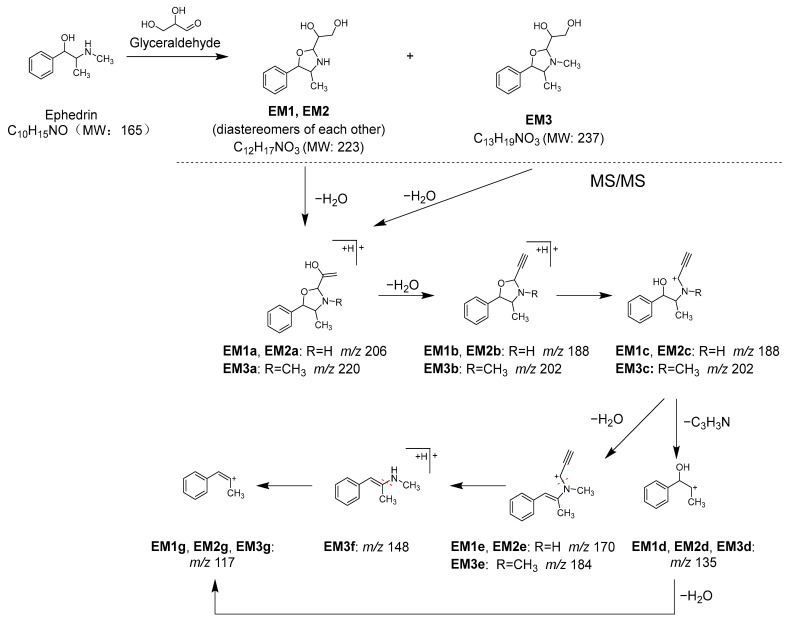
Reaction between ephedrine (**1**) and glyceraldehyde (GA), and MS/MS fragmentation pathway of the reaction products.

To identify the components contributing to the anti-glycation activity, the methanol fraction prepared from Kakkonto was reacted with GA and analyzed using LC-MS (Figure 11A). Comparison of the total ion chromatograms of Kakkonto extract with and without GA addition showed a decrease in the peak intensity of ephedrine (**1**) with GA addition, while three new peaks (**EM1**–**EM3**) were detected. The new peaks were most similar to products generated by the trapping of GA by **1**, as indicated by their molecular formulas of C_12_H_18_O_3_N (**EM1** and **EM2**) and C_13_H_20_O_3_N (**EM3**), which were deduced from the molecular ions [M+H]^+^ observed in MS. Further analysis of MS/MS revealed that **EM1**–**EM3** are derivatives with an oxazoline ring generated through nucleophilic addition reaction and condensation between **1** with GA [41]. In MS/MS of **EM1**–**EM3**, a series of product ions were observed, which were formed through stepwise loss of H_2_O, ring opening, and loss of C_3_H_3_N (Table S2, Figure 12). Since **EM1** and **EM2** showed the same molecular formula and fragment pattern, they were considered to be a pair of diastereomers.

**Figure 13:**
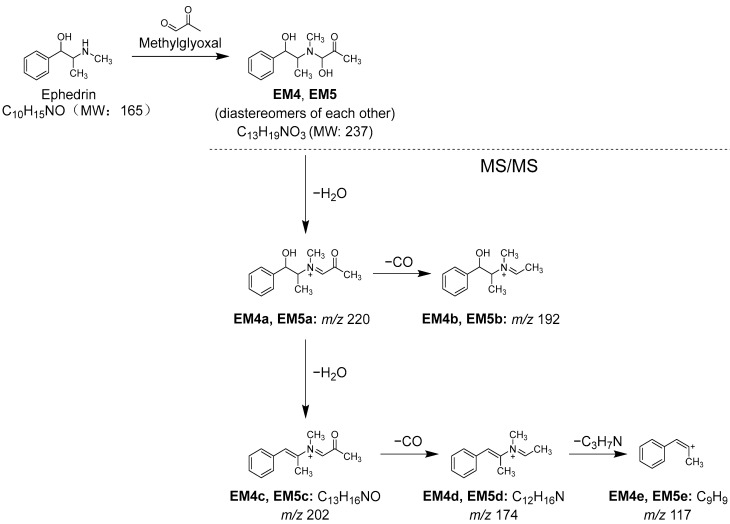
Reaction between ephedrine (**1**) and methylglyoxal (MGO), and MS/MS fragmentation pathway of the reaction products.

Additionally, when the Kakkonto methanol fraction was reacted with MGO and analyzed using LC-MS, a decrease in the peak intensity of ephedrine (**1**) was also observed, along with two new peaks **EM4** and **EM5** (Figure 11B). Peaks **EM4** and **EM5** were identified as a pair of diastereomers formed between **1** and MGO through nucleophilic addition, and their structures were determined based on their molecular formulas, which were obtained from the molecular ions [M+H]^+^ observed in MS, as well as the MS/MS product ions generated through the loss of H_2_O, CO, and C_3_H_7_N (Table S3, Figure 13).

### 2.4. Evaluation of Ephedrae Herba Extract for Anti-Glycation Activity

In Japan, ephedrine (**1**) is a regulated substance by law, which made it impossible to investigate the anti-glycation activity of **1** as a single compound. However, the inhibitory activity of an Ephedrae herba extract, which contained **1** as the main component, was evaluated using the BSA–GA assay. As a result, the extract dose-dependently inhibited glycation in the concentration range of 10–0.125 mg/mL, with an IC_50_ of 0.22 mg/mL (Figure 1B). Its activity was comparable to that of the positive control, aminoguanidine (IC_50_ 2.6 mM, equivalent to 0.19 mg/mL) (Figure 1C). Based on these results, it can be concluded that ephedrine (**1**) is the key component responsible for the anti-glycation activity of Kakkonto, and its possible mechanism of action is trapping the reactive carbonyl species.

So far, there have been no reports on the anti-glycation activity of ephedrine or Ephedra herba, but there have been several reports that they suppress hyperglycemia in vivo. For example, it has been reported that crude extracts of *Ephedra sinica* or *E. foeminea* and *l*-ephedrine showed suppression of hyperglycemia in mice with diabetes induced by streptozotocin [42,43]. Furthermore, extracts of *E. sinica* have been shown to promote the regeneration of atrophied pancreatic islets. It has also been suggested that *E. sinica* could improve hyperglycemia by regenerating atrophied pancreas islets and restoring insulin secretion [42]. In addition, the anti-obesity effect and anti-hyperglycemic action of *Ephedra sinica* were evaluated in mice fed a high-fat diet, and *Ephedra sinica* was found to reduce weight gain and fasting blood glucose levels, as well as improve HDL cholesterol levels [44]. Furthermore, it was suggested that *E. sinica* could improve obesity and hyperglycemia by increasing PPAR-α and adiponectin and decreasing TNF-α [44]. In these reports, it was concluded that the decrease in blood sugar levels was due to improved metabolism, but considering our research results as well, it is possible that the direct trapping of sugar by ephedrine may also be involved.

## 3. Materials and Methods

### 3.1. General Methods

LC-MS analysis was performed on a Vanquish UHPLC system combined with a Q-Exactive Hybrid Quadrupole Orbitrap mass spectrometer. Fluorescence intensity was measured using a multi-label plate reader from EnSpire 2300 (Perkinelmer Japan Co., Ltd., Kanagawa, Japan). A Diaion HP-20 for column chromatography was purchased from Mitsubishi Chemical Co. (Tokyo, Japan).

### 3.2. Materials and Chemicals

A total of 147 oral Kampo prescriptions, which are covered by health insurance in Japan, were purchased from Ohsugi Pharmaceutical Co., Ltd. (Osaka, Japan), Kracie Holdings, Ltd. (Tokyo, Japan), Kotaro Pharmaceutical Co., Ltd. (Osaka, Japan), Sanwa Shoyaku Co., Ltd. (Tochigi, Japan), Taikoseido Pharmaceutical Co., Ltd. (Hyogo, Japan), Tsumura & Co. (Tokyo, Japan), and Toyo-Kampo Pharmaceutical Co., Ltd. (Osaka, Japan), respectively. The Albumin Glycation Assay Kit, Glyceraldehyde (Cat. No. AAS-AGE-K01) was purchased from Cosmo Bio Co., Ltd. (Tokyo, Japan). Sodium azide was purchased from Sigma-Aldrich Japan Co. (Tokyo, Japan). Ephedrae herba was purchased from Tanuma Shokai Co., Ltd. (Chiba, Japan) (Lot. 131017). Methylglyoxal and Glyceraldehyde were purchased from Nacalai Tesque, Inc. (Kyoto, Japan). An amount of 0.1 moL/L Phosphate buffer, acetic acid, and dimethyl sulfoxide were purchased from FUJIFILM Wako Pure Chemical Corporation (Osaka, Japan). LC-MS-grade acetonitrile, methanol, formic acid, and distilled water were purchased from Kanto Chemical Co., Inc. (Tokyo, Japan).

### 3.3. Sample Preparation of Kampo Solutions

We defined the amount of the daily dose as 1 unit (U) for Kampo prescriptions. A Kampo prescription (2 mU) was suspended in 1 mL of purified water and extracted using sonication at room temperature for 15 min. The mixture was then centrifuged at 4800 rpm for 15 min, and the supernatant was used as the 2 mU/mL sample solution.

### 3.4. Assay for the Anti-Glycation Activity Using D-Ribose

An assay of AGE formation inhibitory activity between BSA and D-ribose was conducted according to a method from the literature with minor modifications [22]. The solution containing BSA solution (final concentration 10 mg/mL), each sample solution (final concentration 50 μU/mL), and D-ribose solution (final concentration 0.5 M) were added to each well of a 96-well microplate, and then fluorescence intensity (excitation wavelength: 370 nm, fluorescence wavelength: 440 nm) was measured using a fluorescence plate reader after 1 h of incubation. The glycation value of the vehicle control (0 μU/mL) was assumed to be 100%. The glycation value of each well relative to the control was determined as the glycation ratio.

### 3.5. Assay for the Anti-Glycation Activity Using Glyceraldehyde

The anti-glycation activity using glyceraldehyde was evaluated with an Albumin Glycation Assay Kit, Glyceraldehyde (Cosmo Bio Co., Ltd. (Tokyo, Japan), Cat. No. AAS-AGE-K01). The assay kit buffer was used, with sodium azide (3 mM) added. Each concentration of sample solution was prepared from 2 mU/mL sample solution via dilution using the assay kit buffer to each concentration (final concentrations 400, 200, 100, 50, 25, and 12.5 μU/mL).

BSA solution (50 μL), each concentration sample solution (40 μL), and glyceraldehyde solution (500 mM, 10 μL) were added to each well of a 96-well microplate, and then fluorescence intensity A (excitation wavelength: 370 nm, fluorescence wavelength: 440 nm) was measured using a fluorescence plate reader. After 24 h of incubation, fluorescence intensity B was measured. The glycation value (fluorescence intensity B—fluorescence intensity A) of the vehicle control (0 μU/mL) was assumed to be 100%. The glycation value of each well relative to the control was determined as the glycation ratio.

### 3.6. UHPLC-MS Conditions

UHPLC was conducted using a Vanquish UHPLC system (Thermo Scientific, Waltham, MA, USA). Chromatographic peaks were separated on a TSKgel ODS-120H (100 × 2.0 mm I.D., 1.9 μm) at a flow rate of 0.4 mL/min at 40 °C with a column temperature oven. A mobile phase consisted of eluent A (distilled water with 0.1% formic acid) and B (acetonitrile with 0.1% formic acid) programmed as follows: 0–2.5 min, 10% B, 2.5–15 min,10→35% B, 15–20 min, 35→60% B, 20–25 min, 60→100% B, 25–30 min 100% B, and then the column was re-equilibrated at 10% B for 5 min before the next injection. The injection volume was 2 μL for analysis.

A Q-Exactive hybrid quadrupole orbitrap high-resolution accurate mass spectrometer system (Thermo Scientific, Waltham, MA, USA) with an ESI source was operated in the positive and negative ion modes. The calibration solutions were used to calibrate the ESI-MS to increase mass accuracy. The optimized parameters of mass spectrometry were illustrated below: spray voltage, +3.5 kV (for positive ion mode) or −2.5 kV (for negative ion mode); capillary temperature, 262.5 °C; sheath gas flow rate, 50 units; AUX gas flow rate, 12.5 units; sweep gas flow rate, 2.63 units; S-lens RF level, 50 units; and probe heater temperature, 425 °C. Data were collected in the full MS modes and full MS/data-dependent (dd)-MS/MS. In-source CID was set at 0 eV. The resolution was 70,000 for full MS and 35,000 for full MS/dd-MS/MS. The AGC was set at 1E6 for full MS and 1E5 for dd-MS/MS. Maximum IT was set at 200 ms for full MS. Scan range was set at 150 to 2000 *m/z* for full MS. Data-dependent scan was performed using high-energy collision with normalized collision energy (NCE) at 10 eV; 30 eV; and stepped NCE at 10, 25, and 40 eV.

### 3.7. Preparation of Kakkonto Solution for LC-MS

Kakkonto formulation (2.5 g) was suspended in 25 mL of purified water and extracted via sonication at room temperature for 30 min. The mixture was then centrifuged at 4800 rpm for 15 min, and the supernatant was subjected to Diaion HP-20 column chromatography to yield the water eluted part (300 mL) and methanol eluted part (300 mL). The methanol eluted part was evaporated in vacuo to yield a methanol fraction (0.29 g). The methanol fraction in methanol (1 mg/mL) was filtrated with a 0.22 mm filter and then analyzed using LC-MS.

### 3.8. Evaluation of the GA and MGO Trapping Capacity of Kakkonto

Kakkonto methanol fraction (10 mg) was incubated with GA (50 mM or 0 mM) in PBS buffer (pH 7.4, 100 mM) at 37 °C. After 24 h of incubation, the reaction was stopped by adding 200 μL of acetic acid. Purified water (10 mL) was added to the reaction mixture, followed by Diaion HP-20 column chromatography to yield the water eluted part (100 mL) and the methanol eluted part (100 mL). The methanol eluted part was evaporated in vacuo to yield a methanol fraction. The methanol fraction in 10 mL of methanol was filtrated with 0.22 μm filter and then analyzed using LC-MS. Kakkonto (10 mg) and MGO (50 mM or 0 mM) mixtures were reacted and analyzed using LC-MS using a similar method to that described above.

### 3.9. Extraction and Assay for the Anti-Glycation Activity of Ephedrae Herba

Ephedrae herba (1 g) was suspended in 25 mL of methanol and extracted via sonication at room temperature for 20 min. The mixture was then filtrated, and the filtrate was evaporated in vacuo to yield methanol extract (98 mg). The methanol extract (8 mg) was dissolved with DMSO (80 mL) and 2.5, 1.25, 0.625, and 0.3125 mg/mL of solution (final concentrations 1, 0.5, 0.25, and 0.125 mg/mL) were prepared with the buffer of the Albumin Glycation Assay Kit, Glyceraldehyde. An assay for the anti-glycation activity was evaluated using the same method as that described above.

## 4. Conclusions

In this study, Kakkonto, one of the prescribed Kampo medicines, revealed significant in vitro anti-glycation activity, and ephedrine was identified as the key component responsible for this activity. Kakkonto is clinically used in Japan for the early stages of colds, inflammatory diseases, shoulder stiffness, upper body neuralgia, and hives. Our findings suggest the potential for Kakkonto to be used for the treatment of diabetic complications based on its anti-glycation activity, but further investigation is needed.

## Figures and Tables

**Table 1:** Anti-glycation activity of oral Kampo prescriptions.

		Glycation Inhibitory Ratio (%) ± SE	
No.	Kampo Prescriptions	BSA–D-Ribose Assay	BSA–GA Assay
		50 μU/mL	400 μU/mL
1	Shiniseihaito	111.9 ± 6.2	68.2 ± 0.87
2	Kakkonto	87.5 ± 5.2	111.0 ± 4.0
3	Daisaikotokyodaio	81.8 ± 1.9	60.6 ± 0.46
4	Saikokeishikankyoto	74.0 ± 5.2	54.6 ± 0.64
5	Keishikashakuyakuto	73.0 ± 5.2	55.7 ± 0.9
6	Gorinsan	72.7 ± 6.0	56.2 ± 0.98
7	Sammotsuogonto	71.4 ± 4.4	65.6 ± 1.4
8	Unseiin	71.2 ± 7.0	82.3 ± 1.9
9	Ninjinyoeito	64.6 ± 12.8	<50
10	Saibokuto	62.0 ± 7.3	<50
11	Shosaikoto	62.0 ± 4.2	75.3 ± 12.5
12	Tsudosan	57.4 ± 14.8	89.0 ± 1.1
13	Sansoninto	56.9 ± 5.7	<50
14	Otsujito	56.6 ± 5.6	65.0 ± 1.4
15	Saikanto	55.8 ± 2.9	53.0 ± 6.8
16	Saikokeishito	55.7 ± 9.8	60.6 ± 3.3
17	Hangeshashinto	52.3 ± 2.4	60.4 ± 1.3
18	Bakumondoto	50.1 ± 7.8	<50

## Data Availability

The data presented in this study and samples of the compounds are available upon request from the corresponding author.

## Supplementary Materials

The following supporting information can be downloaded at: https://www.mdpi.com/article/10.3390/molecules28114409/s1. Table S1: Mass and MS/MS spectrometric data of compounds identified in Kakkonto extract. Table S2: Mass spectrometric data of products detected in Kakkonto extract reacted with glyceraldehyde (GA). Table S3: Mass spectrometric data of products detected in Kakkonto extract reacted with methylglyoxal (MGO).

## Sample Availability

Samples of the compounds are available from the authors.
