# Supplementary material for: LC-MS Profiling of Kakkonto and Identification of Ephedrine as a Key Component for Its Anti-Glycation Activity
Source: Molecules. 2023 May 29;28(11):4409. doi: 10.3390/molecules28114409 (PMC10254259; doi:10.3390/molecules28114409)
Supplement: Supplementary file 1 [file molecules-28-04409-s001.zip › molecules-2386738-supplementary.pdf]

Supporting Information

LC-MS Profiling of Kakkonto and Identification of Ephedrine as a Key Component for Its Anti-Glycation Activity

Kaori Ito, Takashi Kikuchi *, Kanako Ikube, Kouharu Otsuki, Kazuo Koike and Wei Li *

Faculty of Pharmaceutical Sciences, Toho University, Miyama 2-2-1, Funabashi 274-8510, Chiba, Japan

* Correspondence: takashi.kikuchi@phar.toho-u.ac.jp (T.K.); liwei@phar.toho-u.ac.jp (W.L.)

Table S1. Mass and MS/MS spectrometric data of compounds identified in Kakkonto extract.

Table S2. Mass spectrometric data of products detected in Kakkonto extract reacted with glyceraldehyde (GA).

Table S3. Mass spectrometric data of products detected in Kakkonto extract reacted with methylglyoxal (MGO).

Table S1. Mass and MS/MS spectrometric data of compounds identified in Kakkonto extract.

			Positive ion mode				Negative ion mode				
			MS		MS^2 (NCE 10 eV)	MS^2 (stepped NCE 10, 25, and 40 eV)	MS		MS^2 (stepped NCE 10, 25, 40 eV)		
No.	t_R (min)	Molecular formula	Detected mass (*m/z*)		Detected mass *m/z* (Intensity, molecular formula)	Detected mass *m/z* (Intensity, molecular formula)	Detected mass (*m/z*)		Detected mass *m/z* (Intensity, molecular formula)	Identification	Source
**1**	1.92	$C_{10}H_{15}ON$	166.1227	$[M+H]^+$	148.1120 (100, $C_{10}H_{14}N$)<br>117.0701 (1.9, C_9H_9)	148.1119 (100, $C_{10}H_{14}N$)<br>117.0701 (4.0, C_9H_9)	no detection			Ephedrine	E
**2**	2.32	$C_{11}H_{17}ON$	180.1383	$[M+H]^+$	180.1382 (100, $C_{11}H_{18}ON$)<br>162.1276 (88.8, $C_{11}H_{16}N$)<br>135.0804 (10.4, $C_9H_{11}O$)<br>117.0700 (2.4, C_9H_9)	162.1277 (100, $C_{11}H_{16}N$)<br>135.0804 (14.3, $C_9H_{11}O$)<br>117.0701 (5.6, C_9H_9)	no detection			Methylephedrine	E
**3**	3.51	$C_{21}H_{20}O_{10}$	433.1127	$[M+H]^+$	433.1125 (100, $C_{21}H_{21}O_{10}$)<br>415.1022 (1.9, $C_{21}H_{19}O_9$)<br>397.0922 (0.2, $C_{21}H_{17}O_8$)<br>367.0809 (0.4, $C_{20}H_{15}O_7$)<br>313.0704 (0.8, $C_{17}H_{13}O_6$)	433.1126 (100, $C_{21}H_{21}O_{10}$)<br>415.1018 (15.9, $C_{21}H_{19}O_9$)<br>397.0912 (17.3,$C_{21}H_{17}O_8$)<br>379.0806 (8.9, $C_{21}H_{15}O_7$)<br>367.0807 (11.1, $C_{20}H_{15}O_7$)<br>337.0701 (12.6, $C_{19}H_{13}O_6$)<br>313.0703 (51.9, $C_{17}H_{13}O_6$)<br>283.0601 (19.6, $C_{16}H_{11}O_5$)	431.0980	$[M–H]^–$	431.0984 (100, $C_{21}H_{19}O_{10}$)<br>311.0563 (80.1, $C_{17}H_{11}O_6$)<br>283.0613 (53.9, $C_{16}H_{11}O_5$)	3'-hydroxypuerarin	PU
**4**	5.52	$C_{21}H_{20}O_9$	417.1176	$[M+H]^+$	417.1174 (100, $C_{21}H_{21}O_9$)<br>399.1071 (2.4, $C_{21}H_{19}O_8$)<br>381.0962 (0.5, $C_{21}H_{17}O_7$)<br>351.0868 (0.4, $C_{20}H_{15}O_6$)<br>321.0761 (0.3, $C_{19}H_{13}O_5$)<br>297.0754 (1.6, $C_{17}H_{13}O_5$)<br>257.0809 (0.9, $C_{15}H_{13}O_4$)	417.1173 (100, $C_{21}H_{21}O_9$)<br>399.1071 (18.2, $C_{21}H_{19}O_8$)<br>381.0965 (4.5, $C_{21}H_{17}O_7$)<br>363.0858 (10.2, $C_{21}H_{15}O_6$)<br>351.0859 (11.6, $C_{20}H_{15}O_6$)<br>335.0908 (4.8, $C_{20}H_{15}O_5$)<br>321.0754 (12.8, $C_{19}H_{13}O_5$)<br>297.0755 (50.3, $C_{17}H_{13}O_5$)<br>281.0808 (3.0, $C_{17}H_{13}O_4$)<br>267.0650 (18.5, $C_{16}H_{11}O_4$)<br>257.0805 (26.4, $C_{15}H_{13}O_4$)	415.1031	$[M–H]^–$	415.1037 (100, $C_{21}H_{19}O_9$)<br>295.0612 (79.6, $C_{17}H_{11}O_5$)<br>267.0665 (58.9, $C_{16}H_{11}O_4$)	Puerarin	PU

**5**	5.76	$C_{23}H_{28}O_{11}$	481.1699	$[M+H]^+$	319.1173 (84.0, $C_{17}H_{19}O_6$)<br>301.1068 (18.9, $C_{17}H_{17}O_5$)<br>197.0808 (100, $C_{10}H_{13}O_4$)<br>179.0701 (2.1, $C_{10}H_{11}O_3$)<br>151.0753 (3.6, $C_9H_{11}O_2$)<br>105.0339 (32.2, C_7H_5O)	319.1172 (23.8, $C_{17}H_{19}O_6$)<br>197.0807 (49.8, $C_{10}H_{13}O_4$)<br>179.0701 (6.6, $C_{10}H_{11}O_3$)<br>151.0753 (19.2, $C_9H_{11}O_2$)<br>133.0648 (19.6, C_9H_9O)<br>105.0338 (100, C_7H_5O)	525.1611	$[M+HCOO]^-$	479.1563 (13.7, $C_{23}H_{27}O_{11}$)<br>121.0294 (100, $C_7H_5O_2$)	Albiflorin	PA
**6**	6.10	$C_{22}H_{22}O_{10}$	447.1282	$[M+H]^+$	447.1280 (100, $C_{22}H_{23}O_{10}$)<br>429.1170 (1.4, $C_{22}H_{21}O_9$)<br>411.1067 (0.4, $C_{22}H_{19}O_8$)<br>381.0955 (0.3, $C_{21}H_{17}O_7$)<br>327.0858 (0.6, $C_{18}H_{15}O_6$)	447.1280 (100, $C_{22}H_{23}O_{10}$)<br>429.1174 (19.8, $C_{22}H_{21}O_9$)<br>411.1068 (18.3, $C_{22}H_{19}O_8$)<br>393.0963 (10.2, $C_{22}H_{17}O_7$)<br>381.0962 (11.8, $C_{21}H_{17}O_7$)<br>365.1011 (5.5, $C_{21}H_{17}O_6$)<br>357.0962 (3.8, $C_{19}H_{17}O_7$)<br>351.0856 (12.0, $C_{20}H_{15}O_6$)<br>337.1063 (7.3, $C_{20}H_{17}O_5$)<br>327.0859 (53.8, $C_{18}H_{15}O_6$)<br>297.0755 (20.1, $C_{17}H_{11}O_5$)	445.1137	$[M–H]^-$	445.1142(100, $C_{22}H_{21}O_{10}$)<br>325.0719 (82.5, $C_{18}H_{13}O_6$)<br>297.0770 (19.4, $C_{17}H_{13}O_5$)	3’-Methoxypuerarin	PU
**7**	6.35	$C_{26}H_{28}O_{13}$	549.1600	$[M+H]^+$	549.1597 (100, $C_{28}H_{29}O_{13}$)<br>417.1171 (5.7, $C_{21}H_{21}O_9$)<br>399.1067 (0.3, $C_{21}H_{19}O_8$)<br>297.0753 (0.4, $C_{17}H_{13}O_5$)	549.1594 (100, $C_{26}H_{29}O_{13}$)<br>417.1171 (60.1, $C_{21}H_{21}O_9$)<br>399.1067 (26.7, $C_{21}H_{19}O_8$)<br>381.0961 (20.8, $C_{21}H_{17}O_7$)<br>351.0856 (13.5, $C_{20}H_{15}O_6$)<br>335.0907 (6.4, $C_{20}H_{15}O_5$)<br>321.0751 (14.7, $C_{19}H_{13}O_5$)<br>297.0753 (62.7, $C_{17}H_{13}O_5$)<br>267.0649 (14.8, $C_{16}H_{11}O_4$)	547.1458	$[M–H]^-$	295.0612 (100, $C_{17}H_{11}O_5$)<br>267.0663 (53.3, $C_{16}H_{11}O_4$)	Puerarin apioside	PU
**8**	6.57	$C_{23}H_{28}O_{11}$	498.1966	$[M+NH_4]^+$	463.1595 (6.0, $C_{23}H_{27}O_{10}$)<br>341.1224 (0.8, $C_{16}H_{21}O_8$)<br>319.1175 (3.3, $C_{17}H_{19}O_6$)<br>301.1069 (13.5, $C_{17}H_{17}O_5$)<br>285.0966 (2.5, $C_{13}H_{17}O_7$)<br>197.0808 (25.2, $C_{10}H_{13}O_4$)<br>179.0701 (100, $C_{10}H_{11}O_3$)	197.0807 (9.4, $C_{10}H_{13}O_4$)<br>179.0701 (100, $C_{10}H_{11}O_3$)<br>151.0753 (26.7, $C_9H_{11}O_2$)	525.1613	$[M+HCOO]^-$	479.1558 (4.1, $C_{23}H_{27}O_{11}$)<br>449.1457 (0.5, $C_{22}H_{25}O_{10}$)<br>327.1088 (8.2, $C_{15}H_{19}O_8$)<br>165.0556 (9.5, $C_9H_9O_3$)<br>121.0294 (100, $C_7H_5O_2$)	Paeoniflorin	PA
**9**	7.20	$C_{21}H_{20}O_9$	417.1175	$[M+H]^+$	255.0650 (100, $C_{15}H_{11}O_4$)	255.0648 (100, $C_{15}H_{11}O_4$)	461.1089	$[M+HCOO]^-$	415.1037 (34.6, $C_{21}H_{19}O_9$)<br>253.0507 (100, $C_{16}H_9O_4$)	Daidzin	PU<br>G
**10**	7.68	$C_{22}H_{22}O_{10}$	447.1284	$[M+H]^+$	285.0755 (100, $C_{16}H_{13}O_5$)	285.0755 (100, $C_{16}H_{13}O_5$)	491.1199	$[M+HCOO]^-$	445.1145 (31.2, $C_{22}H_{21}O_{10}$)<br>283.0613 (100, $C_{16}H_{11}O_5$)	Glycitin	PU

**11**	7.82	$C_{26}H_{28}O_{14}$	565.1550	$[M+H]^+$	565.1548 (100, $C_{26}H_{29}O_{14}$)<br>547.1442 (28.2, $C_{26}H_{27}O_{13}$)<br>433.1128 (29.1, $C_{21}H_{21}O_{10}$)<br>415.1024 (4.4, $C_{21}H_{19}O_9$)<br>313.0703 (3.1, $C_{17}H_{13}O_6$)	565.1548 (100, $C_{26}H_{29}O_{14}$)<br>547.1443 (33.0, $C_{26}H_{27}O_{13}$)<br>397.0910 (56.1, $C_{21}H_{17}O_8$)<br>379.0808 (34.7, $C_{21}H_{15}O_7$)<br>337.0703 (18.4, $C_{19}H_{13}O_6$)<br>325.0703 (22.8, $C_{18}H_{13}O_6$)<br>283.0600 (3.4, $C_{16}H_{11}O_5$)	563.1408	$[M–H]^–$	563.1407 (66.1, $C_{26}H_{27}O_{14}$)<br>473.1092 (5.0, $C_{23}H_{21}O_{11}$)<br>443.0984 (14.1, $C_{19}H_{13}O_7$)<br>353.0667 (5.4, $C_{18}H_{13}O_7$)<br>311.0565 (100, $C_{17}H_{11}O_6$)<br>283.0615 (24.4, $C_{16}H_{11}O_5$)	3'-Hydroxypuerarin apioside	PU
**12**	7.96	$C_{29}H_{34}O_{14}$	607.2022	$[M+H]^+$	571.1796 (1.7, $C_{29}H_{31}O_{12}$)<br>461.1437 (100, $C_{23}H_{25}O_{10}$)<br>299.0912 (65.4, $C_{17}H_{15}O_5$)<br>205.0493 (0.7, $C_{11}H_9O_4$)<br>107.0493 (1.9, C_7H_7O)	461.1436 (46.9, $C_{23}H_{25}O_{10}$)<br>299.0912 (66.0, $C_{17}H_{15}O_5$)<br>281.0807 (11.8, $C_{17}H_{13}O_4$)<br>253.0855 (11.8, $C_{16}H_{13}O_3$)<br>239.0700 (8.6, $C_{15}H_{11}O_3$)<br>107.0495 (100, C_7H_7O)	605.1884	$[M–H]^–$	297.0769 (100, $C_{17}H_{13}O_5$)<br>253.0871 (92.2, $C_{16}H_{13}O_3$)	Pueroside A	PU
**13**	8.50	$C_{21}H_{22}O_9$	436.1597	$[M+NH_4]^+$	419.1331 (4.0, $C_{21}H_{23}O_9$)<br>257.0806 (100, $C_{15}H_{13}O_4$)<br>145.0495 (1.2, $C_6H_9O_4$)<br>137.0233 (2.7, $C_7H_5O_3$)	257.0806 (100, $C_{15}H_{13}O_4$)<br>239.0698 (1.1, $C_{15}H_{11}O_3$)<br>147.0440 (3.5, $C_9H_7O_2$)<br>137.0233 (8.1, $C_7H_5O_3$)<br>97.0289 (1.1, $C_5H_6O_2$)	417.1187	$[M–H]^–$	255.0665 (100, $C_{15}H_{11}O_4$)<br>135.0087 (35.1, $C_7H_3O_3$)<br>119.0501 (18.1, C_8H_7O)	Liquiritin	G
**14**	9.43	$C_{27}H_{30}O_{13}$	563.1760	$[M+H]^+$	563.1751 (100, $C_{27}H_{31}O_{13}$)<br>431.1332 (4.9, $C_{22}H_{23}O_9$)<br>413.1228 (0.5, $C_{22}H_{21}O_8$)<br>311.0923 (0.4, $C_{18}H_{15}O_5$)	563.1752 (100, $C_{27}H_{31}O_{13}$)<br>431.1333 (57.2, $C_{22}H_{23}O_9$)<br>413.1226 (32.0, $C_{22}H_{21}O_8$)<br>395.1122 (22.3, $C_{22}H_{19}O_7$)<br>377.1013 (10.7, $C_{22}H_{17}O_6$)<br>365.1016 (13.3, $C_{21}H_{17}O_6$)<br>349.1064 (5.9, $C_{21}H_{17}O_5$)<br>335.0910 (15.5, $C_{20}H_{15}O_5$)<br>321.1117 (4.8, $C_{20}H_{17}O_4$)<br>311.0911 (71.3, $C_{18}H_{15}O_5$)<br>281.0808 (15.4, $C_{17}H_{13}O_4$)	561.1619	$[M–H]^–$	309.0770 (100, $C_{18}H_{13}O_5$)<br>281.0820 (29.7, $C_{17}H_{13}O_4$)	formononetin 8-*C*-[β-D-apiofuranosyl-(1→6)]-β-D-glucopyranoside	PU

**15**	10.38	$C_{23}H_{28}O_{11}$	481.1704	$[M+H]^+$	319.11743 (32.2, $C_{17}H_{19}O_6$)<br>301.1070 (7.0, $C_{17}H_{17}O_5$)<br>285.1126 (0.6, $C_{17}H_{17}O_4$)<br>267.0864 (3.1, $C_{13}H_{15}O_6$)<br>257.1378 (1.5, $C_{13}H_{21}O_{15}$)<br>249.0751 (0.8, $C_{13}H_{13}O_5$)<br>215.0910 (0.9, $C_{10}H_{15}O_5$)<br>197.0808 (100, $C_{10}H_{13}O_4$)<br>179.0701 (1.8, $C_{10}H_{11}O_3$)<br>161.0597 (0.6, $C_{10}H_9O_2$)<br>151.0753 (1.6, $C_9H_{11}O_2$)<br>145.0495 (0.7, $C_6H_9O_4$)<br>133.0650 (0.4, C_9H_9O)<br>105.0339 (10.3, C_7H_5O)	319.1173 (18.9, $C_{17}H_{19}O_6$)<br>197.0808 (100, $C_{10}H_{13}O_4$)<br>179.0702 (29.6, $C_{10}H_{11}O_3$)<br>161.0596 (14.0, $C_{10}H_9O_2$)<br>151.0753 (14.0, $C_9H_{11}O_2$)<br>133.0648 (27.9, C_9H_9O)<br>105.0338 (71.4, C_7H_5O)	525.1616	$[M+HCOO]^-$	479.1563 (13.4, $C_{23}H_{27}O_{11}$)<br>121.0294 (100, $C_7H_6O_2$)	4-epi-albiflorin	PA
**16**	11.19	$C_{24}H_{26}O_{10}$	475.1598	$[M+H]^+$	313.1067 (100, $C_{18}H_{17}O_5$)<br>295.0968 (1.3, $C_{18}H_{15}O_4$)<br>267.1026 (0.1, $C_{17}H_{15}O_3$)<br>253.0864 (0.2, $C_{16}H_{13}O_3$)<br>219.0650 (4.2, $C_{12}H_{11}O_4$)<br>207.0651 (0.8, $C_{11}H_{11}O_4$)<br>145.0496 (0.8, $C_6H_9O_4$)<br>107.0495 (14.1, C_7H_7O)	313.1066 (64.2, $C_{18}H_{17}O_5$)<br>295.0963 (10.3, $C_{18}H_{15}O_4$)<br>267.1014 (12.7, $C_{17}H_{15}O_3$)<br>253.0857 (7.3, $C_{16}H_{13}O_3$)<br>219.0650 (6.9, $C_{12}H_{11}O_4$)<br>207.0651 (6.5, $C_{11}H_{11}O_4$)<br>189.0546 (1.8, $C_{11}H_9O_3$)<br>175.0754 (2.6, $C_{11}H_{11}O_2$)<br>161.0596 (1.0, $C_{10}H_9O_2$)<br>145.0646 (2.0, $C_{10}H_9O$)<br>127.0391 (2.3, $C_6H_7O_3$)<br>107.0495 (100, C_7H_7O)	519.1508	$[M+HCOO]^-$	473.1453 (2.5, $C_{24}H_{25}O_{10}$)<br>311.0927 (100, $C_{18}H_{15}O_5$)<br>267.1021 (62.5, $C_{17}H_{15}O_3$)	Sophoraside A	PU
**17**	11.60	$C_{22}H_{22}O_9$	431.1334	$[M+H]^+$	269.0806 (100, $C_{16}H_{13}O_4$)	269.0807 (100, $C_{16}H_{13}O_4$)	475.1247	$[M+HCOO]^-$	267.0665 (100, $C_{16}H_{11}O_4$)	Ononin	PU,<br>G
**18**	11.88	$C_{21}H_{22}O_9$	419.1333	$[M+H]^+$	257.0806 (100, $C_{15}H_{13}O_4$)<br>137.0233 (0.4, $C_7H_5O_3$)	257.0806 (100, $C_{15}H_{13}O_4$)<br>147.0440 (7.4, $C_9H_7O_2$)<br>137.0233 (12.7, $C_7H_5O_3$)	417.1192	$[M–H]^-$	255.0664 (100, $C_{15}H_{11}O_4$)<br>135.0088 (30.1, $C_7H_3O_3$)<br>119.0501 (11.6, C_8H_7O)	Isoliquiritin	G
**19**	12.47	$C_{15}H_{10}O_4$	255.0649	$[M+H]^+$	255.0649 (100, $C_{15}H_{11}O_4$)	255.0650 (100, $C_{15}H_{11}O_4$)	253.0506	$[M–H]^-$	253.0506 (100, $C_{15}H_9O_4$)	Daidzein	PU<br>G
**20**	12.71	$C_{15}H_{12}O_4$	257.0807	$[M+H]^+$	257.0805 (100, $C_{15}H_{13}O_4$)<br>239.0703 (1.5, $C_{15}H_{11}O_3$)<br>211.0755 (0.3, $C_{14}H_{11}O_2$)<br>147.0439 (3.2, $C_9H_7O_2$)<br>137.0233 (4.6, $C_7H_5O_3$)	257.0806 (100, $C_{15}H_{13}O_4$)<br>211.0753 (3.2, $C_{14}H_{11}O_2$)<br>163.0389 (1.6, $C_9H_7O_3$)<br>147.0440 (19.3, $C_9H_7O_2$)<br>137.0233 (35.9, $C_7H_5O_3$)	255.1663	$[M–H]^-$	255.0665 (90.9, $C_{15}H_{11}O_4$)<br>135.0088 (90.2, $C_7H_3O_3$)<br>119.0502 (100, C_8H_7O)<br>91.0189 (11.3, C_6H_3O)	Liquiritigenin	G
**21**	12.99	$C_{16}H_{12}O_5$	285.0754	$[M+H]^+$		285.0755 (100, $C_{16}H_{13}O_5$)[a]	283.0612	$[M–H]^-$	283.0613 (100, $C_{16}H_{11}O_5$)	Glycitein	PU

**22**	14.86	$C_{30}H_{32}O_{12}$	602.2230	$[M+NH_4]^+$	567.1841 (7.1, $C_{30}H_{31}O_{11}$)<br>463.1586 (3.1, $C_{23}H_{27}O_{10}$)<br>445.1489 (22.3, $C_{23}H_{25}O_9$)<br>427.1385 (23.1, $C_{23}H_{23}O_8$)<br>301.1068 (18.5, $C_{17}H_{17}O_5$)<br>267.0863 (93.9, $C_{13}H_{15}O_6$)<br>249.0755 (28.4, $C_{13}H_{13}O_5$)<br>197.0809 (1.8, $C_{10}H_{13}O_4$)<br>179.0702 (100, $C_{10}H_{11}O_3$)<br>151.0753 (1.8, $C_9H_{11}O_2$)<br>105.0338 (3.0, C_7H_5O)	267.0862 (62.7, $C_{13}H_{15}O_6$)<br>249.0755 (45.3, $C_{13}H_{13}O_5$)<br>179.0702 (88.6, $C_{10}H_{11}O_3$)<br>151.0753 (30.6, $C_9H_{11}O_2$)<br>105.0338 (100, C_7H_5O)	629.1881	$[M+HCOO]^-$	583.1821 (5.6, $C_{30}H_{31}O_{12}$)<br>121.0294 (100, $C_7H_5O_2$)	Benzoylpaeoniflorin	PA
**23**	15.20	$C_{30}H_{32}O_{12}$	585.1966	$[M+H]^+$	319.1174 (100, $C_{17}H_{19}O_6$)<br>301.1069 (16.1, $C_{17}H_{17}O_5$)<br>267.0863 (28.7, $C_{13}H_{15}O_6$)<br>249.0756 (6.4, $C_{13}H_{13}O_5$)<br>197.0808 (25.3, $C_{10}H_{13}O_4$)<br>151.0753 (1.5, $C_9H_{11}O_2$)<br>105.0339 (18.6, C_7H_5O)	319.1174 (32.3, $C_{17}H_{19}O_6$)<br>301.1069 (6.1, $C_{17}H_{17}O_5$)<br>267.0863 (10.0, $C_{13}H_{15}O_6$)<br>249.0756 (9.6, $C_{13}H_{13}O_5$)<br>197.0808 (18.2, $C_{10}H_{13}O_4$)<br>151.0753 (12.2, $C_9H_{11}O_2$)<br>105.0339 (100, C_7H_5O)	629.1880	$[M+HCOO]^-$	583.1825 (10.4, $C_{30}H_{31}O_{12}$)<br>121.0294 (100, $C_7H_5O_2$)	Paeonivayin	PA
**24**	15.24	$C_{48}H_{72}O_{21}$	985.4637	$[M+H]^+$	985.4625 (100, $C_{48}H_{73}O_{21}$)<br>809.4303 (13.5, $C_{42}H_{65}O_{15}$)<br>615.3882 (26.2, $C_{36}H_{55}O_8$)<br>453.3357 (14.6, $C_{30}H_{45}O_3$)	809.4305 (7.6, $C_{42}H_{65}O_{15}$)<br>647.3774 (3.4, $C_{36}H_{55}O_{10}$)<br>633.3985 (2.1, $C_{36}H_{57}O_9$)<br>615.3884 (30.9, $C_{36}H_{55}O_8$)<br>471.3465 (8.0, $C_{30}H_{47}O_4$)<br>453.3358 (100, $C_{30}H_{45}O_3$)<br>435.3253 (7.4, $C_{30}H_{43}O_2$)<br>407.3302 (6.3, $C_{29}H_{43}O$)<br>389.3197 (2.6, $C_{29}H_{41}$)<br>357.2418 (3.6, $C_{23}H_{33}O_3$)<br>217.1586 (6.3, $C_{15}H_{21}O$)<br>189.1637 (5.7, $C_{14}H_{21}$)	983.4503	$[M–H]^-$	983.4498 (100, $C_{48}H_{71}O_{21}$)<br>821.3972 (24.1, $C_{42}H_{61}O_{16}$)<br>645.3640 (3.5, $C_{36}H_{53}O_{10}$)<br>351.0569 (36.5, $C_{12}H_{15}O_{12}$)<br>289.0569 (2.6, $C_{11}H_{13}O_9$)<br>193.0353 (19.5, $C_6H_9O_7$)<br>175.0247 (9.4, $C_6H_7O_6$)<br>157.0142 (4.0, $C_6H_5O_5$)<br>113.0243 (34.6, $C_5H_5O_3$)	Licorice saponin A_3	G
**25**	16.09	$C_{44}H_{64}O_{18}$	881.4155	$[M+H]^+$	705.3835 (91.6, $C_{38}H_{57}O_{12}$)<br>529.3523 (2.2, $C_{32}H_{49}O_6$)<br>511.3415 (100, $C_{32}H_{47}O_5$)	705.3832 (11.7, $C_{38}H_{57}O_{12}$)<br>529.3522 (5.1, $C_{32}H_{49}O_6$)<br>511.3414 (100, $C_{32}H_{47}O_5$)<br>451.3202 (5.1, $C_{30}H_{43}O_3$)<br>433.3096 (2.3, $C_{30}H_{41}O_2$)<br>405.3148 (3.1, $C_{29}H_{41}O$)<br>233.1536 (1.3, $C_{15}H_{21}O_2$)<br>217.1585 (2.5, $C_{15}H_{21}O$)<br>215.1433 (1.3, $C_{15}H_{19}O$)<br>189.1636 (2.1, $C_{14}H_{21}$)<br>187.1481 (2.5, $C_{14}H_{19}$)	879.4023	$[M–H]^-$	351.0570 (95.6, $C_{12}H_{15}O_{12}$)<br>289.0562 (7.0, $C_{11}H_{13}O_9$)<br>193.0353 (49.9, $C_6H_9O_7$)<br>175.0247 (25.8, $C_6H_7O_6$)<br>157.0140 (5.8, $C_6H_5O_5$)<br>113.0243 (100, $C_5H_5O_3$)	22β-acetoxyglycyrrhizic acid	G

**26**	17.08	$C_{42}H_{62}O_{17}$	839.4055	$[M+H]^+$	663.3727 (21.4, $C_{36}H_{55}O_{11}$)<br>645.3621 (12.9, $C_{36}H_{53}O_{10}$)<br>487.3414 (81.2, $C_{30}H_{47}O_5$)<br>469.3309 (100, $C_{30}H_{45}O_4$)<br>451.3203 (12.5, $C_{30}H_{43}O_3$)	645.3633 (5.7, $C_{36}H_{53}O_{10}$)<br>487.3415 (54.5, $C_{36}H_{53}O_{10}$)<br>469.3311 (100, $C_{30}H_{45}O_4$)<br>451.3203 (23.5, $C_{30}H_{43}O_3$)<br>439.3205 (4.8, $C_{29}H_{43}O_3$)<br>217.1581 (2.5, $C_{15}H_{21}O$)<br>205.1588 (1.4, $C_{14}H_{21}O$)<br>189.1637 (4.5, $C_{14}H_{21}$)<br>175.1482 (3.9, $C_{13}H_{19}$)<br>159.0287 (7.2, $C_6H_7O_5$)<br>141.0181 (20.2, $C_6H_5O_4$)	837.3918	$[M–H]^-$	837.3921 (100, $C_{42}H_{61}O_{17}$)<br>351.0571 (65.6, $C_{12}H_{15}O_{12}$)<br>289.0564 (4.2, $C_{11}H_{13}O_9$)<br>193.0353 (39.6, $C_6H_9O_7$)<br>175.0248 (17.2, $C_6H_7O_6$)<br>157.0141 (4.8, $C_6H_5O_5$)<br>113.0244 (65.4, $C_5H_5O_3$)	Licorice saponin G_2	G
**27**	17.77	$C_{42}H_{62}O_{16}$	823.4097	$[M+H]^+$	647.3777 (31.0, $C_{36}H_{55}O_{10}$)<br>471.3463 (5.4, $C_{30}H_{47}O_4$)<br>453.3356 (100, $C_{30}H_{45}O_3$)	647.3784 (13.8, $C_{36}H_{55}O_{10}$)<br>471.3468 (6.0, $C_{30}H_{47}O_4$)<br>453.3359 (100, $C_{30}H_{45}O_3$)<br>407.3302 (3.2, $C_{29}H_{43}O$)<br>217.1587 (3.2, $C_{15}H_{21}O$)<br>189.1638 (3.3, $C_{14}H_{21}$)	821.3966	$[M–H]^-$	821.3975 (100, $C_{42}H_{61}O_{16}$)<br>351.0572 (65.6, $C_{12}H_{15}O_{12}$)<br>193.0353 (39.6, $C_6H_9O_7$)<br>175.0248 (17.02, $C_6H_7O_6$)<br>157.0141 (4.8, $C_6H_5O_5$)<br>113.0244 (65.4, $C_5H_5O_3$)	Glycyrrhizin	G

PU: PUERARIAE RADIX (*Pueraria lobata*)

E: EPHEDRAE HERBA (*Ephedra sinica*; *Ephedra intermedia*; *Ephedra equisetina*)

PA: PAEONIAE RADIX (*Paenonia lactiflora*)

G: GLYCYRRHIZAE RADIX (*Glycyrrhiza uralensis*; *Glycyrrhiza glabra*)

[a] Normalized collision energy at 30 eV

Table S2. Mass spectrometric data of products detected in Kakkonto extract reacted with glyceraldehyde (GA).

No.	t_R (min)	Molecular formula	Positive ion mode Detected mass (*m/z*)		MS/MS (30 eV) Detected mass *m/z* (Intensity, molecular formula)
**EM1**	1.80	$C_{12}H_{18}O_3N$	224.1281	[M+H]+	206.1174 (100.0, $C_{12}H_{16}O_2N$)
					188.1068 (6.6, $C_{12}H_{14}ON$)
					170.0962 (5.2, $C_{12}H_{12}N$)
					158.0962 (5.5, $C_{11}H_{12}N$)
					134.0963 (5.2, $C_9H_{12}N$)
					117.0699 (0.6, C_9H_9)
**EM2**	2.42	$C_{12}H_{18}O_3N$	224.1280	[M+H]+	206.1174 (100.0, $C_{12}H_{16}O_2N$)
					188.1068 (1.0, $C_{12}H_{14}ON$)
					135.0804 (11.4, $C_9H_{11}O$)
					117.0700 (4.5, C_9H_9)
**EM3**	2.60	$C_{13}H_{20}O_3N$	238.1438	[M+H]+	220.1330 (100.0, $C_{13}H_{18}O_2N$)
					202.1226 (3.0, $C_{13}H_{16}ON$)
					184.1119 (0.9, $C_{13}H_{14}N$)
					172.1119 (1.4, $C_{12}H_{14}N$)
					148.1120 (21.3, $C_{10}H_{14}N$)
					135.0803 (0.6, $C_9H_{11}O$)
					117.0702 (0.4, C_9H_9)
					114.0915 (0.8, $C_6H_{12}ON$)
					104.0709 (4.3, $C_4H_{10}O_2N$)

Table S3. Mass spectrometric data of products detected in Kakkonto extract reacted with methylglyoxal (MGO).

No.	t_R (min)	Molecular formula	Positive ion mode Detected mass *m/z*		MS/MS (30 eV) Detected mass *m/z* (Intensity, molecular formula)
**EM4**	3.07	$C_{13}H_{19}O_3N$	238.1436	[M+H]+	220.1330 (49.2, $C_{13}H_{18}O_2N$)<br>202.1226 (0.6, $C_{13}H_{16}ON$)<br>192.1381 (2.5, $C_{12}H_{18}ON$)<br>174.1274 (0.8, $C_{12}H_{16}N$)<br>135.0804 (17.2, $C_9H_{11}O$)<br>117.0700 (4.6, C_9H_9)<br>104.0709 (100.0, $C_4H_{10}O_2N$)
**EM5**	3.59	$C_{13}H_{19}O_3N$	238.1436	[M+H]+	220.1329 (68.3, $C_{13}H_{18}O_2N$)<br>202.1223 (0.7, $C_{13}H_{16}ON$)<br>192.1380 (3.8, $C_{12}H_{18}ON$)<br>174.1276 (0.9, $C_{12}H_{16}N$)<br>135.0803 (18.6, $C_9H_{11}O$)<br>117.0700 (5.7, C_9H_9)<br>104.0709 (100, $C_4H_{10}O_2N$)
